# Improving urine testing stewardship with a technology-leveraged urine testing guideline

**DOI:** 10.1017/ice.2026.10430

**Published:** 2026-07

**Authors:** Christian J. Ostrowski, Mandy C. Swann, Jacob R. Gillen, Anthony Baffoe-Bonnie

**Affiliations:** 1 Virginia Tech Carilion School of Medicine, Roanoke, VA, USA; 2 Carilion Clinic Roanoke Memorial Hospital, Roanoke, VA, USA

## Abstract

**Background:**

Unnecessary urine cultures contribute to inappropriate antibiotic use, antimicrobial resistance, and *Clostridioides difficile* infection, particularly when asymptomatic bacteriuria (ASB) is misclassified as infection. We evaluated the diagnostic stewardship impact of an algorithm-based best practice alert (BPA) embedded in the electronic medical record (EMR) to guide urine testing in hospitalized adults.

**Methods:**

This prospective quality improvement study was conducted at a 740-bed tertiary care hospital. The BPA triggered when urinalysis with reflex to culture (UACC) was ordered for patients admitted ≥48 hours, guiding clinicians through an algorithm-based ordering workflow. Monthly rates of UACC and pan-culturing were compared between a 12-month pre-intervention period and a 12-month post-intervention period using interrupted time series (ITS) and Mood’s median two-sample test. Gram-negative rod (GNR) bacteremia rates were monitored for safety.

**Results:**

Urine testing decreased from 6.45 to 4.41 tests per 1,000 patient-days (31.6% reduction; *P* < .01), and pan-culturing decreased from 3.47 to 2.70 per 1,000 patient-days (22.2% reduction; *P* < .01). ITS showed declining trends both before and after implementation, without significant immediate changes in level or slope following the intervention. CAUTI rates remained stable (0.91 vs 0.82 per 1,000 catheter-days; *P* = .68), as did rates of gram-negative rod bacteremia (0.47 vs 0.70 per 1,000 patient-days; *P* = .22). Algorithm adherence averaged 63.9% and increased over time (*P* < .01). CAUTI cases classified as potential asymptomatic bacteriuria declined from 31.8% to 25.0% (*P* = .68).

**Conclusions:**

An EMR-integrated, algorithm-based BPA coincided with sustained lower urine testing and pan-culturing rates without adverse safety signals within the context of existing downward trends. Ongoing monitoring is needed to sustain adoption and appropriate use.

## Introduction

Urinary tract infections (UTIs) are among the most common healthcare-associated infections (HAIs) and represent a significant clinical and financial burden.^1^ Diagnosing UTIs in hospitalized patients can be challenging, as symptoms often overlap with other conditions. Fever, dysuria, suprapubic discomfort, or costovertebral tenderness may suggest infection, but nonspecific findings, particularly in older adults, frequently lead to uncertainty.^2^ Asymptomatic bacteriuria (ASB) complicates this picture. ASB is common in hospitalized patients, with prevalence ranging from 15% among women aged 65–80 to nearly 100% in those with long-term indwelling catheters.^3,4^ Outside of specific situations—such as pregnancy, recent urologic procedures, or early postrenal transplant—treatment of ASB offers no clinical benefit and exposes patients to unnecessary antimicrobial therapy. Such overtreatment drives antimicrobial resistance and increases the risk of *Clostridioides difficile* infection.^5,6^

Diagnostic stewardship plays a key role in delivering evidence-based care and reducing waste. Reducing unnecessary urine testing—particularly testing that reflexively leads to urine culturing—is therefore a key target for diagnostic stewardship, with likely beneficial effects on antibiotic prescribing. Recent guidelines have clarified the role of diagnostic stewardship in reducing the chances of misidentification of ASB as a symptomatic infection.^7^ Prior studies have shown that best practice alerts (BPAs) within the electronic medical record (EMR) can influence ordering behavior, particularly in patients with urinary catheters.^8–10^ Additionally, studies done within the Veteran’s Affairs Healthcare system have shown promise in utilizing clinical support tools to improve stewardship in both the inpatient and outpatient setting.^11–13^ However, there remains a need for scalable interventions that address both catheterized and non-catheterized patients, integrate decision support seamlessly into clinician workflows, and monitor safety outcomes.

To address this gap, we developed a clinical decision support tool in the form of a standardized, algorithm-based guideline for urine testing and embedded it within an Epic Systems EMR as a BPA. The goal of this study was to evaluate the impact of this intervention on urine testing and pan-culture rates, while monitoring for potential safety concerns such as increases in gram-negative (GNR) bacteremia rates. We hypothesized that usage of the BPA would be associated with a reduction in urine testing and pan-culture episodes, thereby improving increasing urine-related diagnostic stewardship at our institution.

## Methods

### Study setting and design

This prospective study was conducted at a 740-bed tertiary care teaching hospital in Southwest Virginia. The intervention was implemented across 20 adult inpatient medical and surgical units, including intensive care, intermediate care, and general floor units. Pediatric, labor and delivery, and mother/baby units were excluded from the intervention, and an additional five adult units that participated in a pilot of the intervention were excluded from this analysis. The intervention launched in June 2023. Data from a 12-month pre-intervention period (June 2022–May 2023) and a 12-month post-intervention period (August 2023–July 2024) were compared, with June and July 2023 serving as washout months.

### Definitions

Urinalysis with Reflex to Culture (UACC): A provider-ordered urinalysis that, if predefined laboratory criteria are met, automatically triggers a reflex urine culture according to institutional protocols. UACC orders represent the primary clinician decision point for initiating urine diagnostic evaluation and were the primary target of the clinical decision support intervention.

Urine Culture (UC): A stand-alone provider order for urine culture without an accompanying urinalysis. Institutional ordering policies limit routine access to stand-alone urine culture orders.

Urine Testing: A composite term representing provider-initiated urine diagnostic ordering behavior during the study period, inclusive of UACC orders and directly ordered urine cultures (UCs). Reflex urine cultures generated by the laboratory following UACC orders were not counted as separate urine testing event.

Pan-culture: Defined for this study as the ordering of cultures from two or more anatomic sites within the same calendar day, with at least one order involving urine testing (defined as a UACC or a directly ordered UC).

Ordering Rate: The monthly number of urine testing events or pan-culture events per 1,000 patient-days.

Length of Stay (LOS): The median number of days a patient was admitted to a study unit.

Catheter-Associated Urinary Tract Infection (CAUTI): Defined using National Healthcare Safety Network (NHSN) surveillance criteria.^14^

Potential Asymptomatic Bacteriuria (ASB): CAUTI cases that met NHSN criteria based only on fever, with a documented alternative source of infection unrelated to the urinary tract (e.g., pneumonia).

### Study implementation

**Figure 1.**
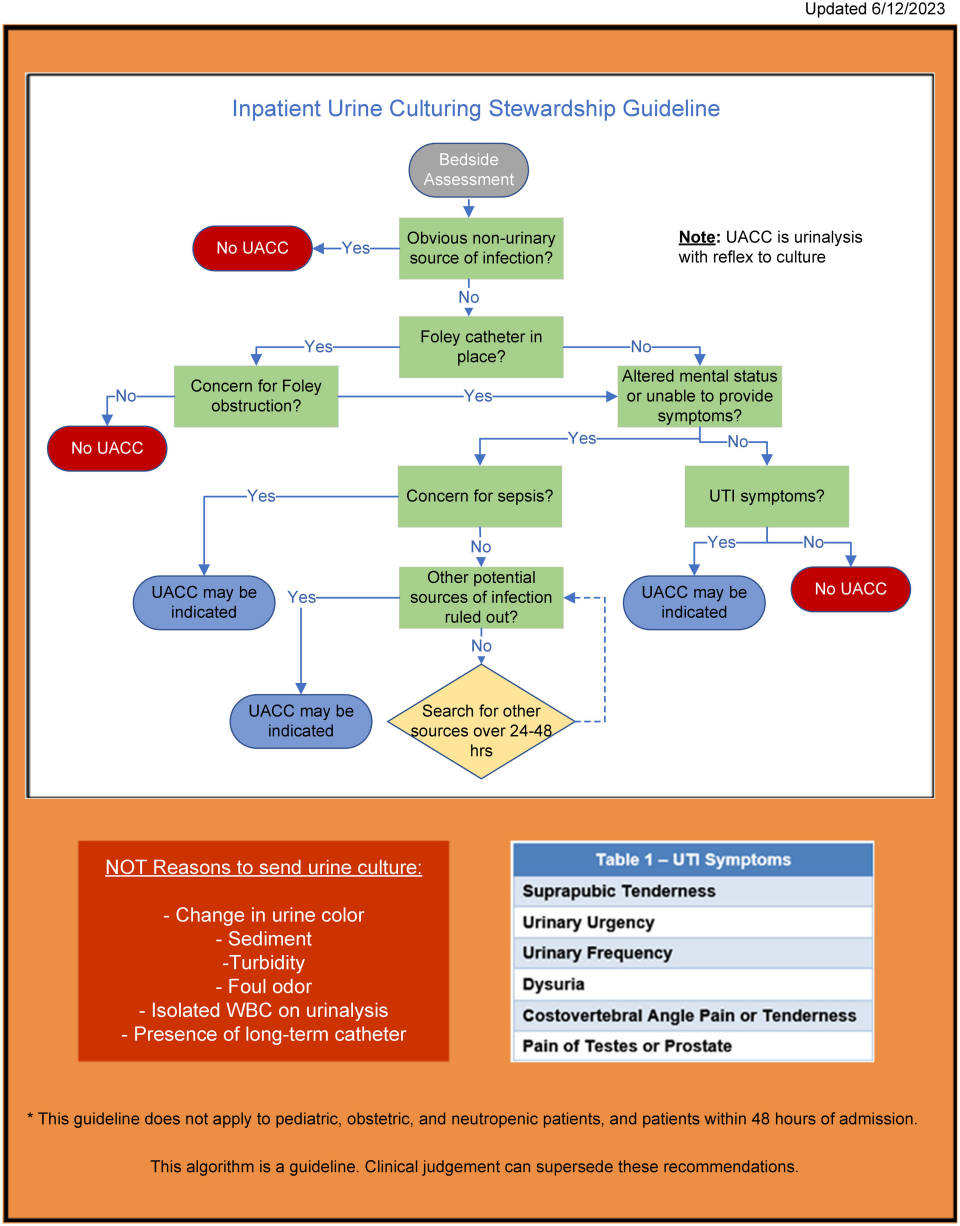
Figure 1 long description. Screenshot of the algorithm that served as the basis for the best practice alert.

An interdisciplinary team of clinical and operational experts developed a decision-tree algorithm to guide evaluation of suspected UTIs. Informed by best practices and literature, the algorithm included guidance for screening and managing ASB in patients with indwelling catheters.^3,15^ A schematic of the algorithm is shown in Figure 1. To support successful adoption, the intervention included educational initiatives focused on distinguishing ASB in those with indwelling catheters from symptomatic UTI and promoting appropriate urine testing.

The decision-tree algorithm was integrated into the provider ordering workflow as a best practice alert (BPA) in Epic. The BPA fired whenever a provider attempted to order a UACC for a patient admitted for ≥48 hours. When triggered, the BPA displayed the algorithm and prompted the provider to select the appropriate algorithm path to justify the order or enter a free-text rationale if the clinical situation was not reflected in the algorithm. Providers also had the option to cancel the order if it was determined to be non-indicated. The BPA fired consistently across all adult inpatient units included in the study.

Longstanding institutional ordering policies limited routine access to stand-alone UC orders. Therefore, the BPAs were overwhelmingly triggered by UACC orders. Direct UC orders account for a small minority of BPA firings (Table S1).

### Outcome measures

The primary study outcome was the monthly number of urine testing orders per 1,000 patient days. Secondary outcomes included the monthly number of pan-cultures per 1,000 patient days, CAUTIs per 1,000 urinary catheter-days, patient LOS, and rates of GNR and *Enterococcus* spp. bacteremia per 1,000 patient days. Monitoring GNR and *Enterococcus* bacteremia served as a safety metric to identify potential under-culturing that could delay diagnosis or lead to urosepsis.

### Adherence monitoring and feedback

Algorithm adherence was assessed monthly. Every fifth urine testing order underwent manual chart review to evaluate clinical rationale and classify orders as on-algorithm or off-algorithm. All reported CAUTI cases also underwent chart review. Cases meeting NHSN CAUTI criteria based solely on fever were evaluated for alternative non-urinary sources of infection at the time of urine testing and classified as potential ASB. Audit findings, including algorithm adherence and identification of potential ASB among reported CAUTI cases, were shared with providers and hospital leadership to maintain awareness, reinforce education, and support sustained engagement with the intervention.

### Data collection

Patient demographic and clinical data, including age, sex, BMI, and comorbidity rates, were extracted from the EMR for all eligible patients during the study period.

### Statistical analysis

Interrupted time series analyses were conducted to evaluate changes in urine testing and pan-culturing rates following the intervention, using monthly data from 12 months before and after implementation. For descriptive pre- and post-intervention comparisons, Mood’s median two-sample test was used to compare median rates of urine testing, pan-culturing, CAUTI, and GNR bacteremia. LOS was compared using a paired t-test. *χ*^2^ tests were used to compare patient demographic characteristics and rates of potential misidentification of asymptomatic bacteriuria among patients with indwelling urinary catheters.

Trends in algorithm adherence over time were assessed using the Mann–Kendall trend test. All analyses were conducted using R version 4.5.0, with a two-sided *P* value < .05 considered statistically significant.

## Results

Within the study units there were no significant differences in patient gender, race, or BMI between the pre- and post-intervention periods (Table 1). There were statistically significant differences in patient age, the proportion of patients with diabetes mellitus, and total LOS; however, the absolute differences in these metrics were small (Table 1).

**Table 1.** Comparison of study population demographics before and after the implementation of the intervention

	Pre-Intervention (24,165 patient-days)	Post-Intervention (28,564 patient-days)	*p*-value
Age – median (IQR)	66.66 (22.08)	67.19 (22.02)	<.01*
Sex			.374
Female	12,253 (50.7%)	13,370 (50.3%)	
Male	11,912 (49.3%)	13,205 (49.7%)	
Race			.956
Black	3,044 (12.6%)	3,328 (12.5%)	
White	20,230 (83.7%)	22,259 (83.8%)	
Other/Unknown	891 (3.7%)	988 (3.7%)	
BMI - median (IQR)	28.31 (9.9)	28.18 (9.8)	.0955
Diabetes			.0279*
No	15,506 (64.2%)	16,802 (63.2%)	
Yes	8,659 (35.8%)	9,773 (36.8%)	
Total LOS – mean (SD)	6.09 (3.7)	5.56 (2.9)	<.01*

BMI, body mass index; LOS, length of stay.

**Figure 2.**
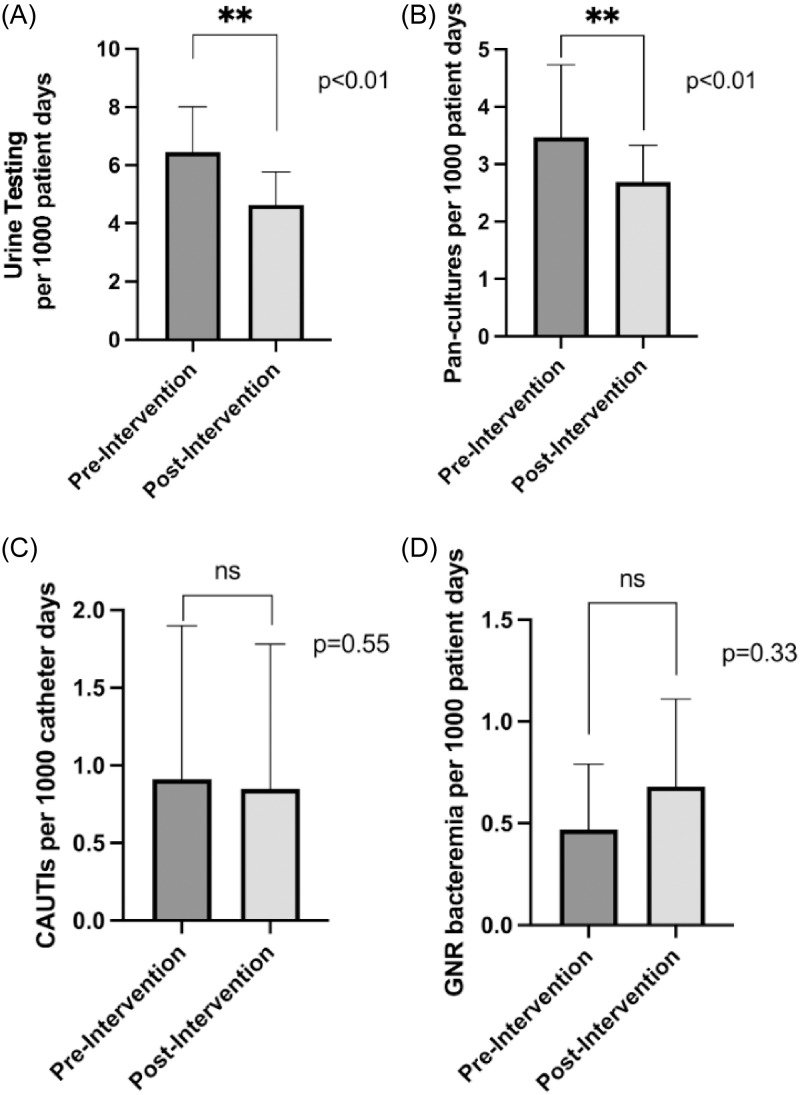
A) comparison of urine testing before and after the implementation of the best practice alert (BPA), B) comparison of pan-culture ordering before and after the implementation of the best practice alert (BPA), C) comparison of catheter associated urinary tract infections (CAUTIs) before and after the implementation of the best practice alert (BPA), D) comparison of gram negative rod (GNR) and enterococcus bacteremia before and after the implementation of the best practice alert (BPA).

**Figure 3.**
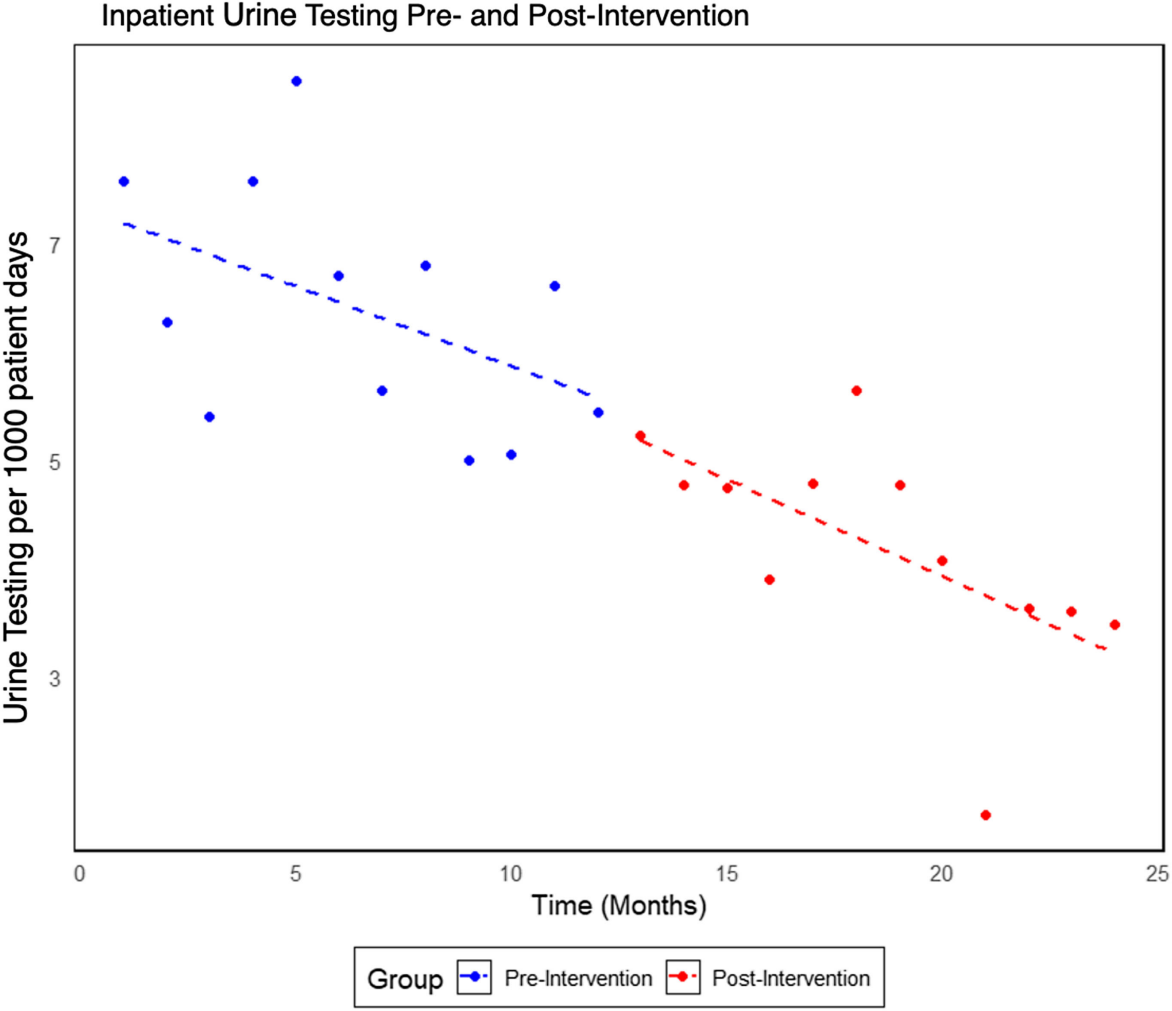
Time series analysis of inpatient urine testing.

**Figure 4.**
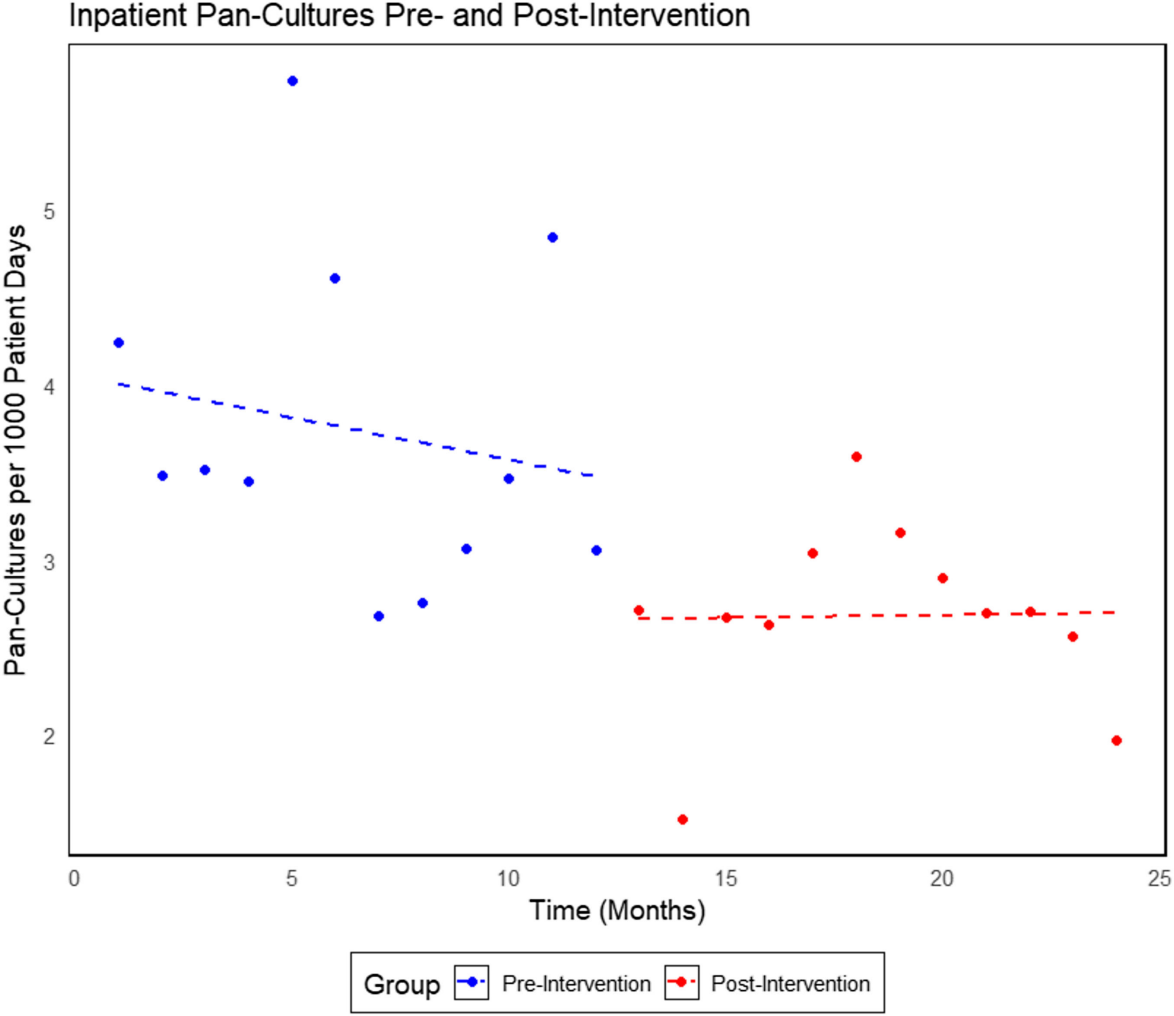
Time series analysis of inpatient pan-culture ordering.

The rate of urine testing in the baseline period was 6.45 tests per 1,000 patient days. During the intervention period, the rate of urine testing decreased significantly to 4.41 tests per 1,000 patient days, representing a 31.6% decrease (*P* < .01) (Figure 2A). In the time series analysis, the pre-intervention trend demonstrated a non-significant gradual decline in monthly urine tests (*P* = .08). The intervention did not produce a statistically significant immediate change in urine testing (*P* = .78), nor was there evidence of a significant change in slope in the post-intervention period (*P* = .78) (Figure 3). The pan-culture rate decreased from 3.47 pan-cultures per 1,000 patient days to 2.70 cultures per 1,000 patient days during the post-intervention period. This represented a 22.2% decrease (*P* < .01) (Figure 2B). In the time series analysis, the pre-intervention trend was slightly negative but not statistically significant (*P* = .47). The intervention did not result in a significant immediate change in pan-culturing (*P* = .22), nor was there evidence of a significant change in slope during the post-intervention period (*P* = .58) (Figure 4).

No significant change in CAUTI rates was observed, with rates of 0.91 infections per 1,000 catheter-days during the pre-intervention period and 0.82 infections per 1,000 catheter-days post-intervention (*P* = .68) (Figure 2C).

Similarly, the rate of gram-negative rod and Enterococcus bacteremia was 0.47 per 1,000 patient-days before the intervention and 0.70 per 1,000 patient-days afterward, with no statistically significant difference between periods (*P* = .22) (Figure 2D).

**Figure 5.**
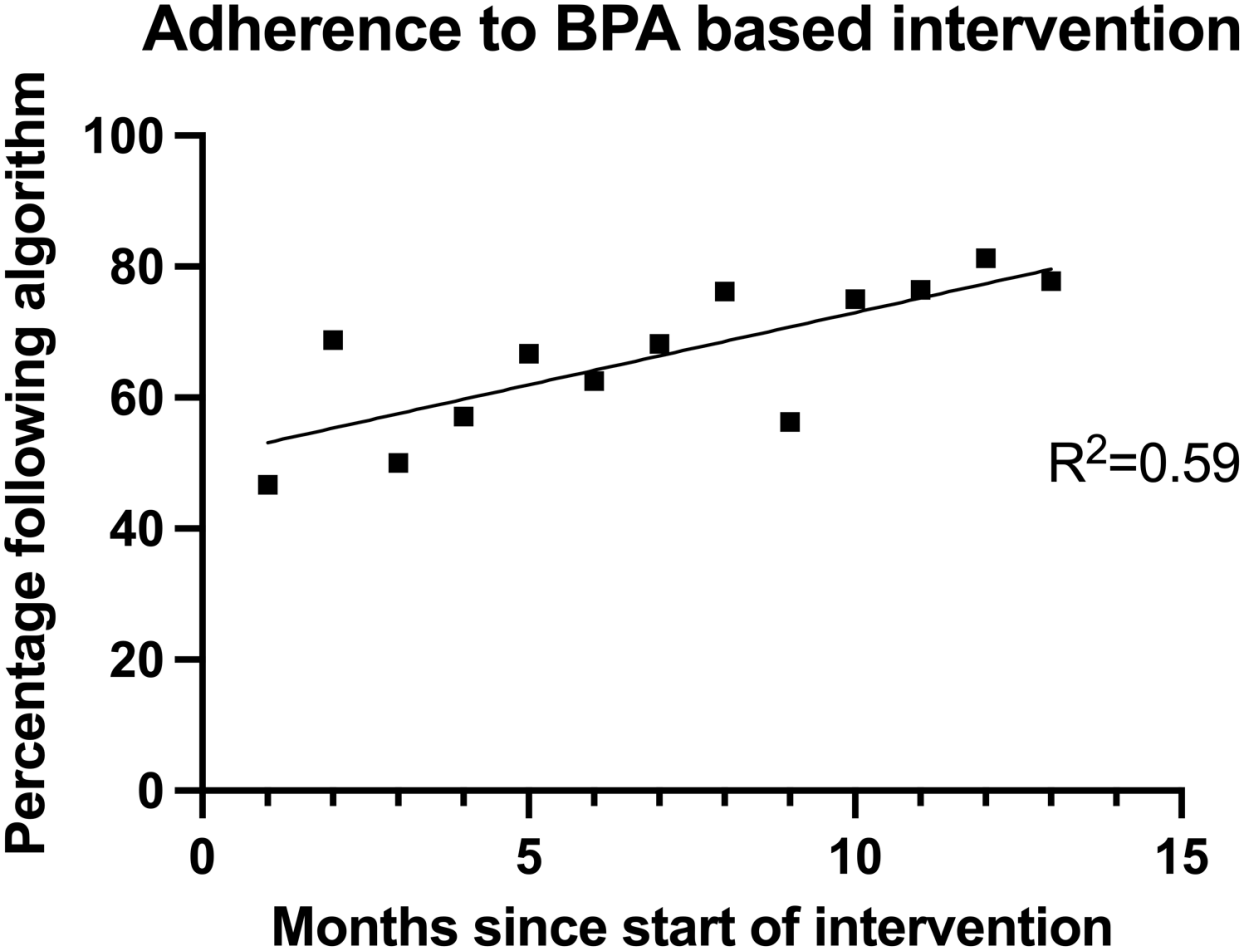
Adherence to the best practice alert(BPA) as measured by chart review.

A total of 222 urine testing orders were reviewed for adherence to the urine testing algorithm. Of these, 142 (63.9%) were classified as on-algorithm and consistent with institutional guidance. Algorithm adherence increased over the intervention period, with a significant upward trend observed over time (*P* < .01) (Figure 5).

Among patients with indwelling urinary catheters, the proportion of reported CAUTI cases classified as potential ASB—defined as cases meeting CAUTI criteria based solely on fever with a concurrent alternative source of infection—was 31.8% in the pre-intervention period and 25.0% in the post-intervention period; this difference was not statistically significant (*P* = .68).

## Discussion

This quality improvement initiative evaluated a multi-component diagnostic stewardship intervention that combined an EMR-integrated, algorithm-based urine testing decision support tool with regular structured clinician feedback across adult inpatient units in a large tertiary care hospital. Over the implementation period, lower urine testing and pan-culturing rates were observed and sustained, without evidence of adverse safety signals.

Several design and implementation features of the intervention help account for these findings. In contrast to many prior diagnostic stewardship interventions, this initiative extended beyond catheterized patients, providing a unified stewardship framework for our adult inpatient population. A key implementation feature was structured adherence monitoring with regular feedback to clinical teams and unit leadership. Overall adherence to the BPA averaged 63.9% and increased steadily over the intervention period, suggesting progressive uptake of the decision-support tool. Persistent off-algorithm orders were most often observed in complex or atypical clinical scenarios not fully addressed by the decision tree, highlighting opportunities for iterative refinement of the algorithm and continued provider education. Future enhancements could incorporate additional clinical pathways or more flexible decision logic to better accommodate these nuanced cases.

Our findings align with a growing body of literature demonstrating the use of EMR-integrated decision support in improving diagnostic stewardship related to urine testing and culturing. Prior studies have shown meaningful reductions in urine culture ordering when clinical decision support requires providers to actively specify an indication at the time of ordering, particularly among catheterized patients.^8–13^ Krouss *et al*. reported significant reductions in urine cultures across 11 hospitals using a BPA that prompted indication selection, while Yarrington *et al*. observed reductions in CAUTI rates and LOS following implementation of a catheter-focused BPA combined with education and feedback.^8,10^ In contrast, other investigations have demonstrated more modest or inconsistent effects, particularly in inpatient settings. Claeys *et al*. found that ordering-based decision support alone was less effective than conditional reflex urine culturing across a three-hospital system, and Stensgard *et al*. reported significant reductions in outpatient urine culture ordering but no corresponding reduction in inpatient ordering.^11,12^

Consistent with expert guidance,^7^ our findings highlight that diagnostic stewardship interventions often exert incremental effects that depend on baseline practices, intervention design, and clinical context. The overall number of cultures was lower in the post-intervention period. However, the interrupted time series showed a downward trend that predated the intervention and was non-significant for both immediate effect and slope. As a result, it is not clear whether the lower cultures seen in the post-intervention period are directly attributable to the intervention.

In addition to evaluating ordering outcomes, we incorporated surveillance GNR and Enterococcus bacteremia as safety indicators. The observed stability of bacteremia rates suggests that reductions in urine testing did not result in missed diagnoses or delayed treatment, providing additional reassurance regarding the safety of the intervention. However, given the non-significant increase observed, continued monitoring will be important to confirm these findings over longer periods.

### Limitations

This study has several limitations. First, the absence of a contemporaneous control group limits the ability to fully account for secular trends in urine testing and infection outcomes. In addition, the relatively short duration of the study may have contributed to the null findings in the ITS analyses; continued evaluation of the intervention could yield additional insights about possible longer-term effects. Notably, in a prior pilot of this intervention conducted on five inpatient units, substantial reductions in urine testing (41.0%) and pan-culturing (42.2%) were observed on intervention units, with no change in concurrent control units,^16^ providing supportive—though not definitive—evidence that the BPA intervention may have contributed to the reductions observed during the current broader implementation. This was a single-center study conducted within a specific institutional and EMR context, which may limit generalizability. Finally, although algorithm adherence improved over time, residual off-algorithm ordering persisted, particularly in complex clinical scenarios not fully captured by the decision tree.

## Conclusions

Implementation of an algorithm-based BPA coincided with sustained lower urine testing and pan-culturing rates, without increases in GNR or Enterococcus bacteremia in a single-center inpatient setting. It is unclear how much of the observed change can be attributed to the intervention due to preexisting downward trends. Our findings suggest that EMR-integrated diagnostic stewardship may safely augment urine stewardship efforts in the adult inpatient setting. Continued monitoring and iterative refinement will be important to maintain effectiveness, and extended evaluation in diverse care environments is needed to confirm generalizability.

## Supporting information

10.1017/ice.2026.10430.sm001 Ostrowski et al. supplementary material Ostrowski et al. supplementary material

## Supplementary material

The supplementary material for this article can be found at https://doi.org/10.1017/ice.2026.10430.

## Figure 1 Long description

A flowchart for inpatient urine culturing stewardship guideline. The flowchart begins with a bedside assessment. If there is no UACC and an obvious non-urinary source of infection is present, no further action is taken. If there is no obvious non-urinary source of infection, the flowchart checks if a Foley catheter is in place. If there is a concern for Foley obstruction and no Foley catheter is in place, UACC may be indicated. If a Foley catheter is in place and there is a concern for obstruction, UACC may be indicated. If there is no concern for obstruction and the mental status is altered or the patient is unable to provide symptoms, the flowchart checks for concern for sepsis. If there is concern for sepsis, UACC may be indicated. If there is no concern for sepsis, other potential sources of infection are ruled out. If other potential sources of infection are ruled out, UACC may be indicated. If other potential sources of infection are not ruled out, a search for other sources over 24-48 hours is conducted. If there is no altered mental status or inability to provide symptoms, the flowchart checks for UTI symptoms. If UTI symptoms are present, UACC may be indicated. If UTI symptoms are not present, no UACC is taken.

Navigate back to Figure 1.

## References

[1] Hollenbeak CS , Schilling AL. The attributable cost of catheter-associated urinary tract infections in the United States: a systematic review. Am J Infect Control 2018;46:751–757. doi: 10.1016/j.ajic.2018.01.015. 29478760

[2] Tambyah PA , Maki DG. Catheter-associated urinary tract infection is rarely symptomatic: a prospective study of 1,497 catheterized patients. Arch Intern Med 2000;160:678–682. doi: 10.1001/archinte.160.5.678. 10724054

[3] Nicolle LE , Gupta K , Bradley SF , et al. Clinical practice guideline for the management of asymptomatic bacteriuria: 2019 update by the Infectious Diseases Society of Americaa. Clin Infect Dis 2019;68:e83–e110. doi: 10.1093/cid/ciy1121. 30895288

[4] Luu T , Albarillo FS. Asymptomatic bacteriuria: prevalence, diagnosis, management, and current antimicrobial stewardship implementations. Am J Med 2022;135:e236–e244. doi: 10.1016/j.amjmed.2022.03.015. 35367448

[5] Hensgens MPM , Goorhuis A , Dekkers OM , Kuijper EJ. Time interval of increased risk for Clostridium difficile infection after exposure to antibiotics. J Antimicrob Chemother 2012;67:742–748. doi: 10.1093/jac/dkr508. 22146873

[6] CDC. Taking antibiotics increases your risk for *C. diff* infection. Centers for Disease Control and Prevention, 2022. https://www.cdc.gov/cdiff/risk.html. Accessed April 10, 2024.

[7] Fabre V , Davis A , Diekema DJ , et al. Principles of diagnostic stewardship: a practical guide from the Society for Healthcare Epidemiology of America Diagnostic Stewardship Task Force. Infect Control Hosp Epidemiol 2023;44:178–185. doi: 10.1017/ice.2023.5. 36786646

[8] Krouss M , Alaiev D , Shin DW , et al. Choosing wisely initiative for reducing urine cultures for asymptomatic bacteriuria and catheter-associated asymptomatic bacteriuria in an 11-hospital safety net system. Am J Infect Control 2023;51:461–465. doi: 10.1016/j.ajic.2023.01.005. 36870917

[9] Kalorin CM , Dixon JM , Fike LV , et al. Reducing catheter-associated urinary tract infections across a hospital system through urine culture stewardship. Mayo Clin Proc Innov Qual Outcomes 2022;6:488–495. doi: 10.1016/j.mayocpiqo.2022.08.004. 36176423 PMC9512841

[10] Yarrington ME , Reynolds SS , Dunkerson T , et al. Using clinical decision support to improve urine testing and antibiotic utilization. Infect Control Hosp Epidemiol 2023;44:1582–1586. doi: 10.1017/ice.2023.30. 36987849 PMC10539479

[11] Stensgard E , Masoud B , Gravely A , Drekonja D. Clinical decision support menu for reducing unnecessary urine cultures. Antimicrob Steward Healthc Epidemiol ASHE 2024;4:e91. doi: 10.1017/ash.2024.47. 38807934 PMC11131005

[12] Claeys KC , Brown CH , Pineles L , et al. Implementation of diagnostic stewardship to improve urinary tract infection antibiotic use across 3 medical centers. *clin infect dis* 2025;ciaf411. doi: 10.1093/cid/ciaf411. 40720743

[13] Grigoryan L , Naik AD , Lichtenberger P , et al. Analysis of an antibiotic stewardship program for asymptomatic bacteriuria in the veterans affairs health care system. JAMA Netw Open 2022;5:e2222530. doi: 10.1001/jamanetworkopen.2022.22530. 35877123 PMC9315417

[14] Gould CV , Umscheid CA , Agarwal RK , Kuntz G , Pegues DA , Healthcare Infection Control Practices Advisory Committee. Guideline for prevention of catheter-associated urinary tract infections 2009. Infect Control Hosp Epidemiol 2010;31:319–326. doi: 10.1086/651091. 20156062

[15] O’Grady, NP , Barie, PS , Bartlett, JG , et al. Guidelines for evaluation of new fever in critically ill adult patients: 2008 update from the American College of Critical Care Medicine and the Infectious Diseases Society of America. Crit Care Med 2008;36:1330–1349. doi: 10.1097/CCM.0b013e318169eda9. 18379262

[16] Swann M , Lucas A , Ostrowski C , et al. It takes a village: leveraging a multidisciplinary team and technology for urine culturing stewardship. Antimicrob Steward Healthc Epidemiol ASHE 2024;4:s82–s83. doi: 10.1017/ash.2024.217.

