# Supplementary material for: Improving urine testing stewardship with a technology-leveraged urine testing guideline
Source: Infect Control Hosp Epidemiol. 2026 Apr 28;47(7):697–702. doi: 10.1017/ice.2026.10430 (PMC13315540; doi:10.1017/ice.2026.10430)
Supplement: Ostrowski et al. supplementary material [file S0899823X26104309sup001.docx]

| Urine Test Order Type | Pre-Intervention Period | Post-Intervention Period |
| --- | --- | --- |
| Direct urine culture orders(UC) | 60 | 41 |
| Urinalysis with reflex to culture orders(UACC) | 738 | 490 |

Supplementary Table 1. Distribution of Urine Testing Orders by Order Type During the Study Period
